# Whole Body Vibration Attenuates Brain Damage and Neuroinflammation Following Experimental Traumatic Brain Injury

**DOI:** 10.3389/fcell.2022.847859

**Published:** 2022-04-07

**Authors:** Tao Chen, Wen-Bo Liu, Xu Ren, Yun-Fei Li, Wei Li, Chun-Hua Hang, Yu-Hai Wang

**Affiliations:** ^1^ Department of Neurosurgery, The 904th Hospital of PLA, Medical School of Anhui Medical University, Wuxi, China; ^2^ Department of Neurosurgery, Drum Tower Hospital, Medical School of Nanjing University, Nanjing, China; ^3^ Central Laboratory of the First Affiliated Hospital, Weifang Medical University, Weifang, China

**Keywords:** traumatic brain injury, whole body vibration, neuroinflammation, GFAP, Iba-1

## Abstract

Traumatic brain injury (TBI) is still a major public health problem worldwide, and the research of neuroprotective drugs has encountered great difficulties. Whole body vibration (WBV) is a safe and powerful rehabilitative intervention in various clinical settings, but its effect on neurological diseases is not well documented. In this study, we investigated the effects of WBV pretreatment on brain damage following experimental TBI mimicked by controlled cortical impact (CCI) in mice. C57BL/6 J male mice were expose to WBV at 30 Hz twice per day for 20 days and injured by CCI. WBV had no effect on animal body weight, but significantly reduced the TBI-induced brain edema in the cortex. The results of immunostaining showed that the activation of microglia and astrocytes induced by TBI in brain sections was attenuated by WBV. In consistent, WBV markedly inhibited the expression of pro-inflammatory cytokines, while increased the levels of anti-inflammatory cytokine interleukin 10 (IL-10). In addition, WBV pretreatment alleviated neuronal apoptosis in the cortex and suppressed the cleavage of the apoptotic executive molecule caspase-1. The neurological dysfunction following TBI was determined by open field test and Morris Water Maze (MWM) assay. The results showed that motor activity, learning and memory ability were preserved by WBV compared to TBI-injured mice. In summary, our present data identified WBV as a clinically potent strategy with which to attenuate TBI-related brain damage through regulating neuroinflammation.

## Introduction

Traumatic brain injury (TBI), caused by external forces, such as fall, assault or motor vehicle crash, is still a major public health problem, leading to approximately 40% of trauma-related deaths (Jiang et al., 2019; Fakhoury et al., 2021). Due to the high mortality and disability rate, it is estimated that TBI costs more than 400 billion dollars globally every year (Maas et al., 2017). The majority of TBI survivors have worse health outcomes than the general population, and only a quarter of them get long-term functional independence (Ahmed et al., 2017). Thus, TBI prevention programs have been launched by many countries to reduce the injury itself and to mitigate the severity of related medical and social consequences (Fatuki et al., 2020).

Physical exercises, such as walking, running or swimming, have been shown to exert positive effects on physical and cognitive health. Whole body vibration (WBV) with low amplitude and low frequency is an effective and safe way to train the musculoskeletal and nervous system (Seidel and Heide, 1986; Boerema et al., 2018). It is commonly targeted at individuals with limited mobility and walking difficulties. Accumulating evidence shows that WBV is a powerful rehabilitative intervention in various clinical settings, including the treatment of patients with lumbar disk disease, chronic obstructive pulmonary disease (COPD), osteoporosis, osteoarthritis and type 2 diabetes (Bovenzi, 1999; Verschueren et al., 2004; Gloeckl et al., 2012; Kitamoto et al., 2021). More recently, WBV was shown to have beneficial effects on some neurological disorders. Post-ischemic WBV with 40 Hz for 30 days reduced brain damage, inhibited inflammation and improved functional outcomes after ischemic stroke mimicked by transient middle cerebral artery occlusion (MCAO) (Raval et al., 2018). Zhao et al. found that 4 weeks of low amplitude WBV training protects dopaminergic neurons in a mouse model of Parkinson’s disease (PD) (Zhao et al., 2014). In addition, even in healthy young adults with high cognitive functioning, passive WBV was shown to have positive acute effects on attention and inhibition (Regterschot et al., 2014). However, the effect of WBV on brain damage and neurological disfunction following TBI has not been determined. In this study, we investigated the effects of WBV on brain injury and neuroinflammation in a mouse model of experimental TBI.

## Materials and Methods

### Animals

C57BL/6 J male mice (6–8 weeks) were purchased from the Animal Experimental Center of Anhui Medical University. All animal procedures were approved and supervised by the Animal Ethics Committee of Anhui Medical University.

### WBV Treatment and TBI Model

WBV was induced by a vibration machine (Deca Precision Measuring Instruments, Shenzheng, China), and the animals were expose to WBV at 30 Hz twice per day for 20 days. The *in vivo* TBI in mice was mimicked by the controlled cortical impact (CCI) model as previously described (Luo et al., 2019).

### Evaluation of Brain Edema

Brain edema was determined with the wet-dry method at 72 h following TBI as previously described (Chen et al., 2017).

### Immunostaining in Brain Sections

Brain sections with 4 μm thickness were treated with 0.1% Triton X-100, and then were blocked by 5% BSA. The samples were incubated with the following primary antibodies: Iba-1 (1:100, #17198, Cell Signaling), GFAP (1:100, #80788, Cell Signaling), and NeuN (1:500, #24307, Cell Signaling) at 4 °C overnight. After being washed by PBST for three times, the samples were incubated with the secondary antibodies at 37°C for 1 h. TUNEL staining was performed by the standard method according to the manufacturer’s protocol (Roche, Penzberg, Germany). Then, incubation with DAPI was performed to stain the nuclei, and the images were obtained using a Leica SP5 II confocal microscope.

### Enzyme-Linked Immunosorbent Assay

Brain samples were used for ELISA. The levels of the inflammatory cytokines, including IL-1β, IL-6, TNF-α and IL-10, were measured by ELISA kits following the manufacturer’s protocols (Anoric-Bio, Tianjin, China).

### Open Filed Test

The animals were placed in a transparent chamber with infrared sensors. The mice were monitored for 1 h, during which the first 30 min was excluded as the acclimatization period, and the data were analyzed using the Versamax software (Accuscan Instruments, Columbus, OH).

### Morris Water Maze Assay

The MWM test was used to evaluate learning and memory function of mice. The spatial acquisition trial was performed from 6 to 9 days, and the probe trial was performed at 10 days following TBI as previously described (Desai et al., 2021). Mice were gently released into a pool 120 cm in diameter filled with water and mixed with white non-toxic tempera paint. The trial ended when the mouse managed to locate the submerged platform. In case the mouse did not find the platform within 90 s, it was gently guided to the platform. Four trials were given daily from 6 to 9 days for learning. On the 10th day, the platform was removed, and mice were allowed to swim for 60 s.

### Western Blot Assay

A standard western blot assay was performed using the following primary antibodies: cleaved caspase-1 (sc-398715, Santa Cruz, 1:50) and β-actin (ab8226, Abcam, 1:2000). After incubation with secondary antibodies for 1 h, the bands were visualized by using chemiluminescent detection system.

### Statistical Analysis

Statistical analysis was performed using SPSS 16.0, a statistical software package. Student’s t test (one-tailed for western blot and ratio quantification, two-tailed for the others) was performed for all statistical significance. A value of *p* < 0.05 was considered statistically significant.

## Results

### Experimental Flow and Set Up

**FIGURE 1:**
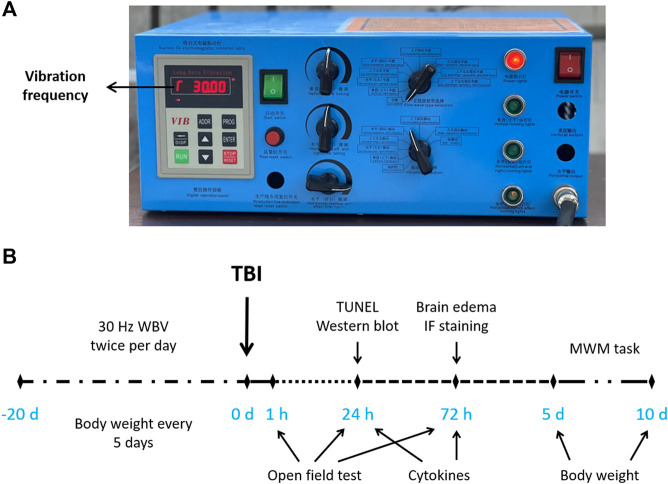
Equipment and experimental design. **(A)** Vibration parameters shown in the vibration controller. **(B)** Experimental design showing the treatments and time points of various measurements.

As shown in Figure 1A, WBV was induced by a vibration machine (Deca Precision Measuring Instruments, Shenzheng, China). As shown in Figure 1B, animals were treated with 30 Hz WBV (30 min per session, two sessions per day, for a period of 20 days), and exposed to TBI. Various measurements were performed at indicated time points.

### WBV Alleviates Brain Damage After TBI

**FIGURE 2:**
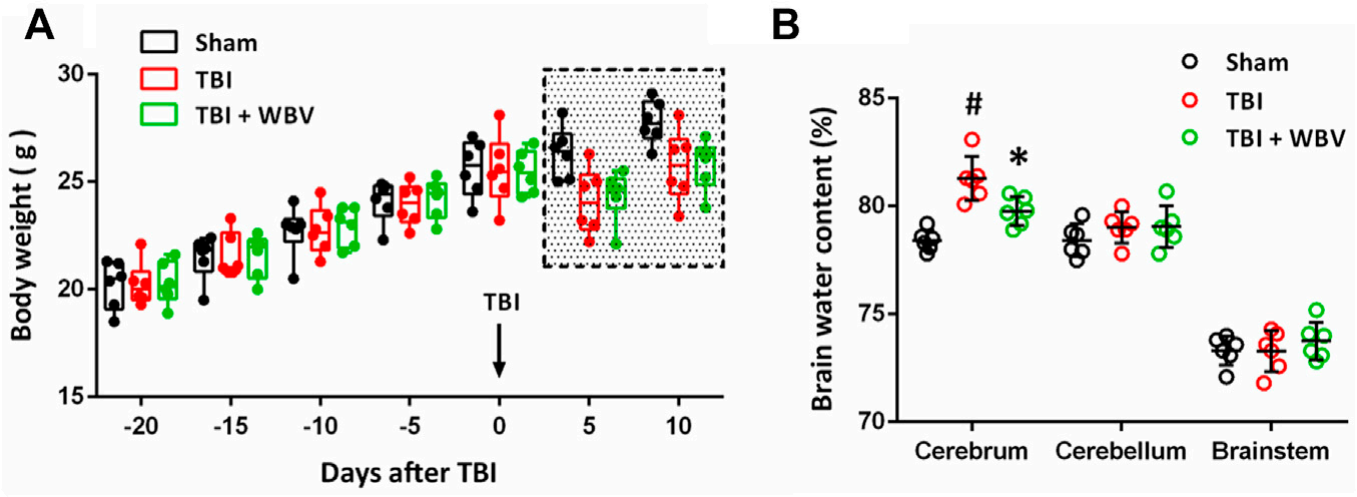
WBV alleviates brain damage after TBI. **(A)** Longitudinal assessment of animals’ body weight. **(B)** Brain edema measurement in cerebrum, cerebellum or brainstem. Data are shown as mean ± SD. ^#^ *p* < 0.05 vs. Sham group. *p < 0.05 vs. TBI group.

Animal body weight was regularly measured for 30 days, and the full longitudinal study data pattern was shown in Figure 2A. We observed that WBV exposure negatively affect body weight. TBI induced a decrease in body weight, whereas there was no difference between TBI and TBI + WBV group at 5 and 10 days after TBI. Brain water content was assayed at 72 h after TBI to determine the effect of WBV on traumatic brain edema (Figure 2B). TBI resulted in a significant increase in brain water content in cerebrum, but not in cerebellum and brainstem. The TBI-induced cortical edema was attenuated by WBV pretreatment.

### WBV Suppresses Iba-1 Expression After TBI

**FIGURE 3:**
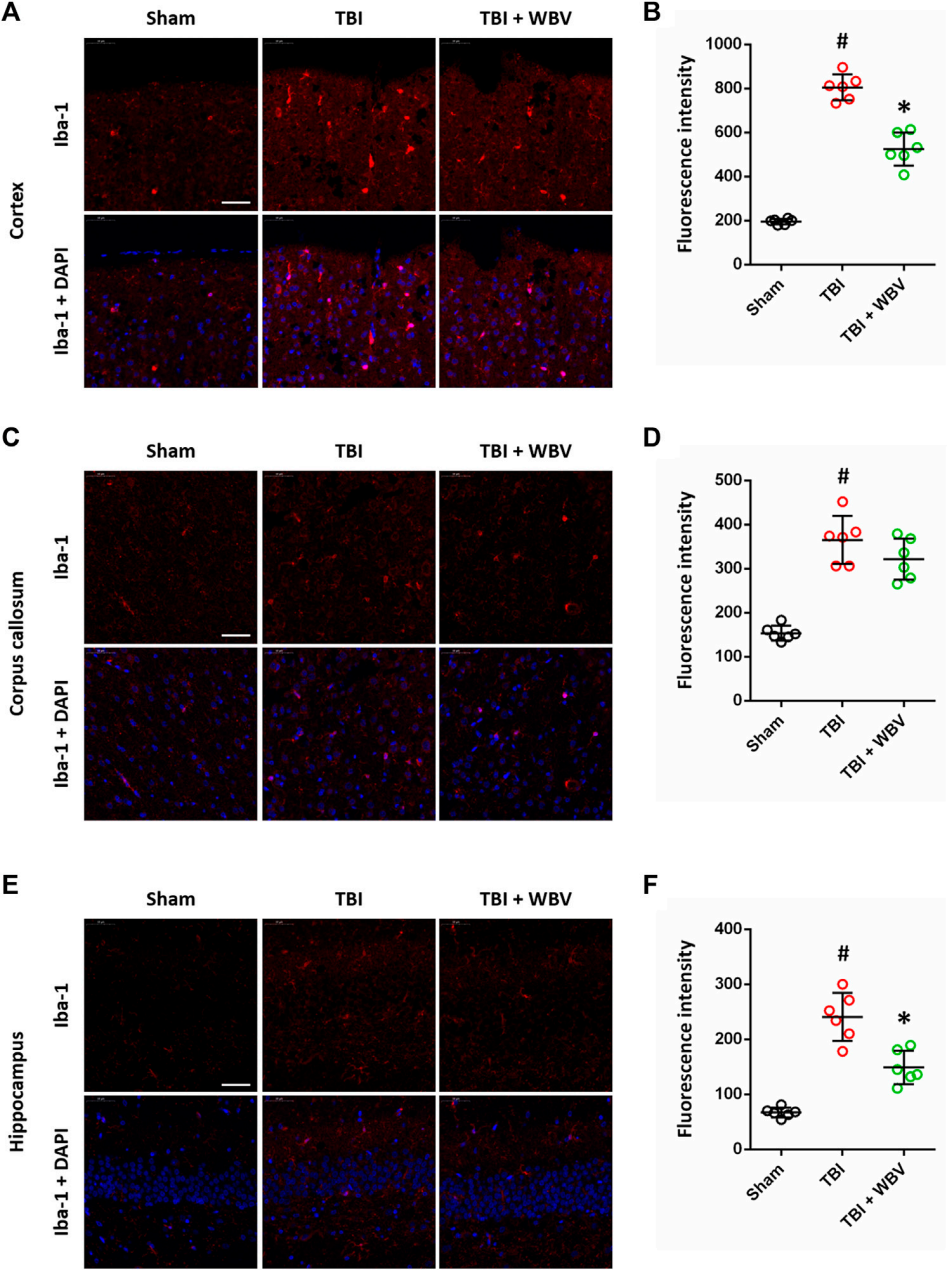
WBV suppresses Iba-1 expression after TBI. **(A,B)** Representative images of Iba-1 immunostaining in cortex **(A)** and quantitative analysis **(B)**. **(C,D)** Representative images of Iba-1 immunostaining in corpus callosum **(C)** and quantitative analysis **(D)**. **(E,F)** Representative images of Iba-1 immunostaining in hippocampus **(E)** and quantitative analysis **(F)**. Scale bar = 50 μm. Data are shown as mean ± SD. ^#^ *p* < 0.05 vs. Sham group. *p < 0.05 vs. TBI group.

We performed immunostaining using Iba-1 antibody at 72 h to detect the activation of microglia in brain sections (Figures 3A,C,E). The TBI-induced increase in Iba-1 expression in cortex (Figure 3B) and hippocampus (Figure 3F) were inhibited by WBV. In corpus callosum, TBI significantly increased Iba-1 expression, which had not been altered by WBV (Figure 3D).

### WBV Inhibits GFAP Expression After TBI

**FIGURE 4:**
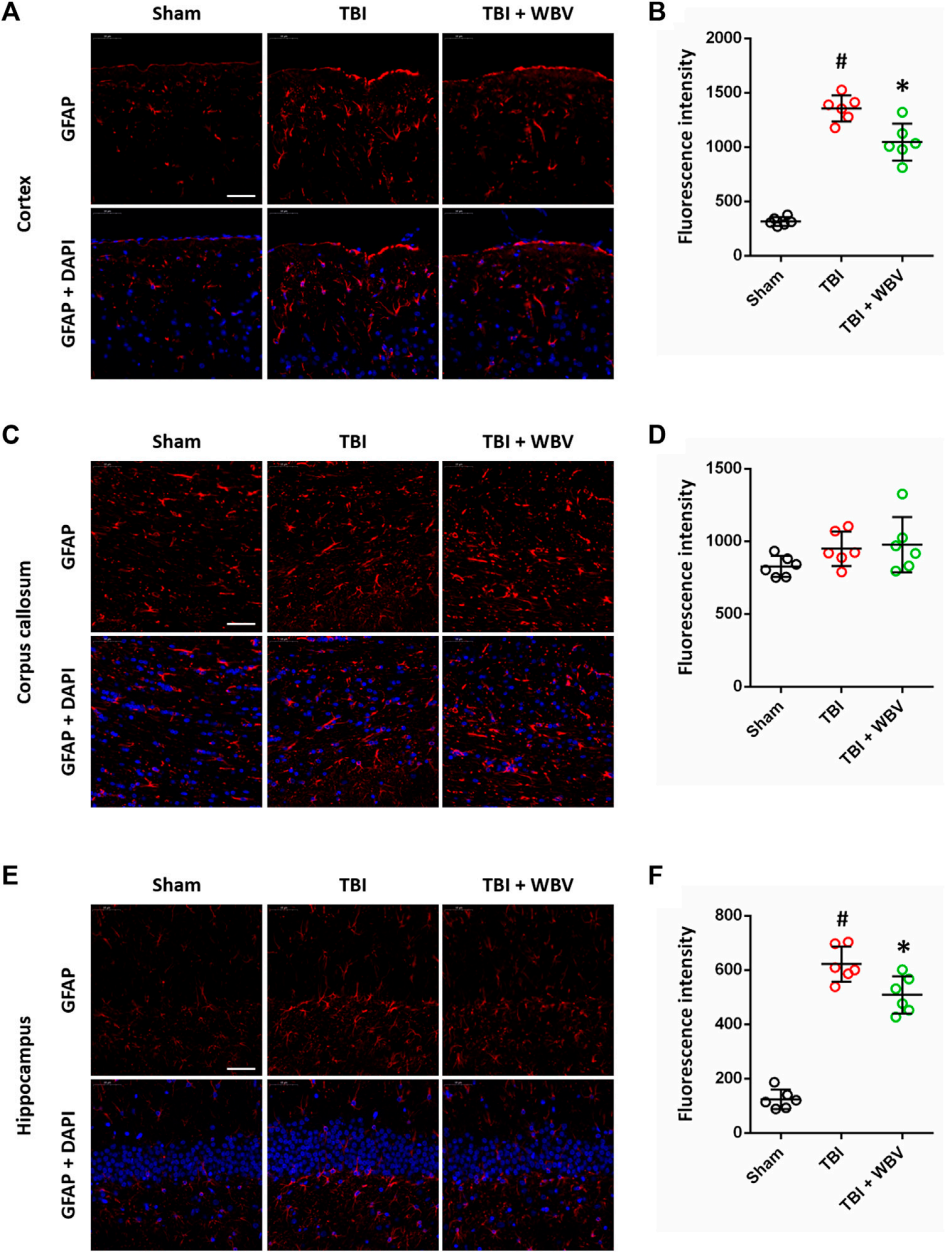
WBV inhibits GFAP expression after TBI. **(A,B)** Representative images of GFAP immunostaining in cortex **(A)** and quantitative analysis **(B)**. **(C,D)** Representative images of GFAP immunostaining in corpus callosum **(C)** and quantitative analysis **(D)**. **(E,F)** Representative images of GFAP immunostaining in hippocampus **(E)** and quantitative analysis **(F)**. Scale bar = 50 μm. Data are shown as mean ± SD. ^#^ *p* < 0.05 vs. Sham group. *p < 0.05 vs. TBI group.

We also performed immunostaining using GFAP antibody at 72 h to detect the activation of astrocytes in brain sections (Figures 4A,C,E). The TBI-induced increase in GFAP expression in cortex (Figure 4B) and hippocampus (Figure 4F) were inhibited by WBV. In corpus callosum, neither TBI nor WBV had effect on GFAP expression (Figure 3D).

### WBV Regulates Inflammatory Cytokines After TBI

**FIGURE 5:**
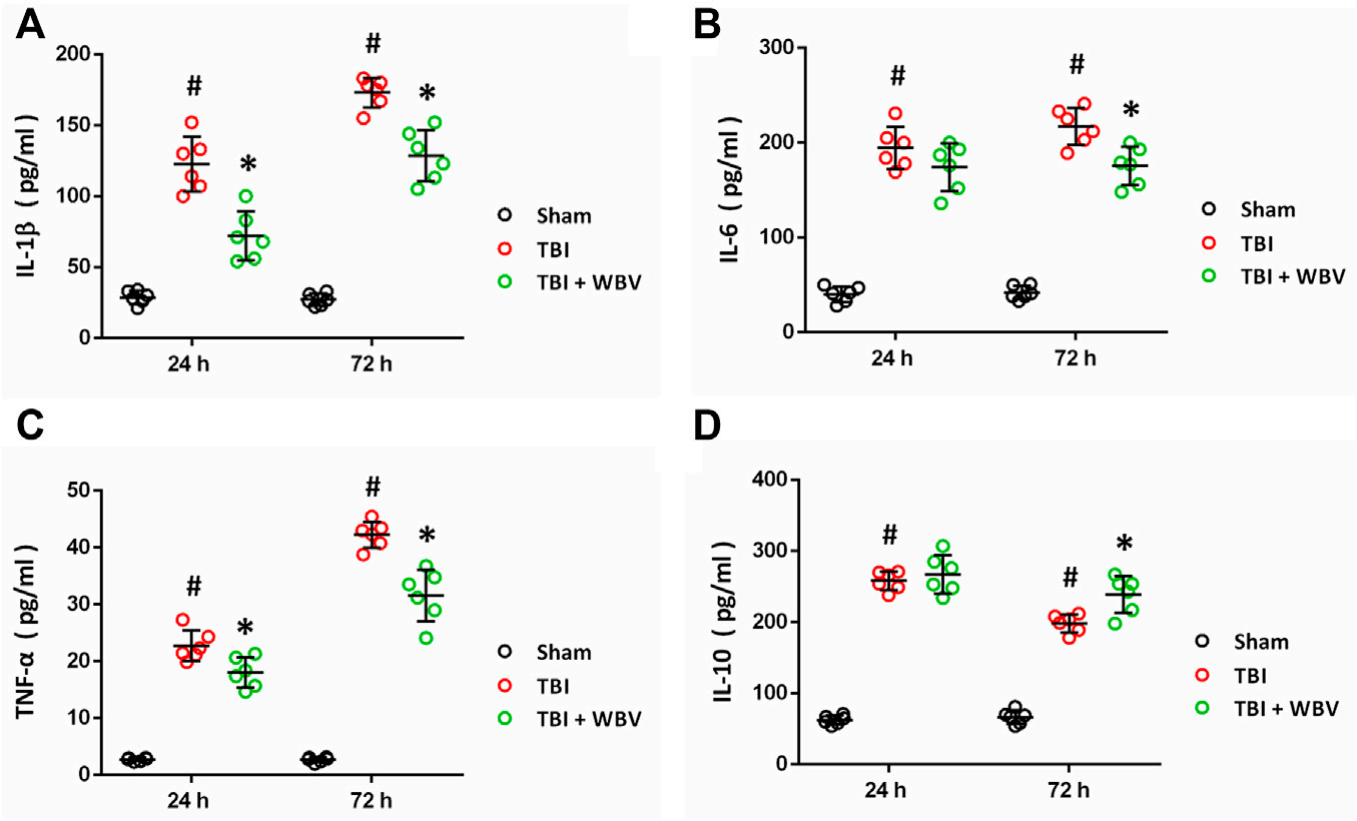
WBV regulates inflammatory cytokines after TBI. **(A–D)** ELISA results of levels of inflammatory cytokines at 24 and 72 h after TBI: IL-1β **(A)**, IL-6 **(B)**, TNF-α **(C)** and IL-10 **(D)**. Data are shown as mean ± SD. ^#^ *p* < 0.05 vs. Sham group. *p < 0.05 vs. TBI group.

ELISA assay was performed to detect the expression of inflammatory cytokines following TBI (Figures 5A–D). The TBI-induced increase in IL-1β (Figure 5A) and TNF-α (Figure 5C) at 24 and 72 h after TBI were partially prevented by WBV. The TBI-induced increase in IL-6 (Figure 5B) at 24 h, but not at 72 h, were attenuated by WBV. In contrast, the TBI-induced increase in IL-10 (Figure 5D) at 24 h, but not at 72 h, were further enhanced by WBV.

### WBV Ameliorates Neuronal Cell Death After TBI

**FIGURE 6:**
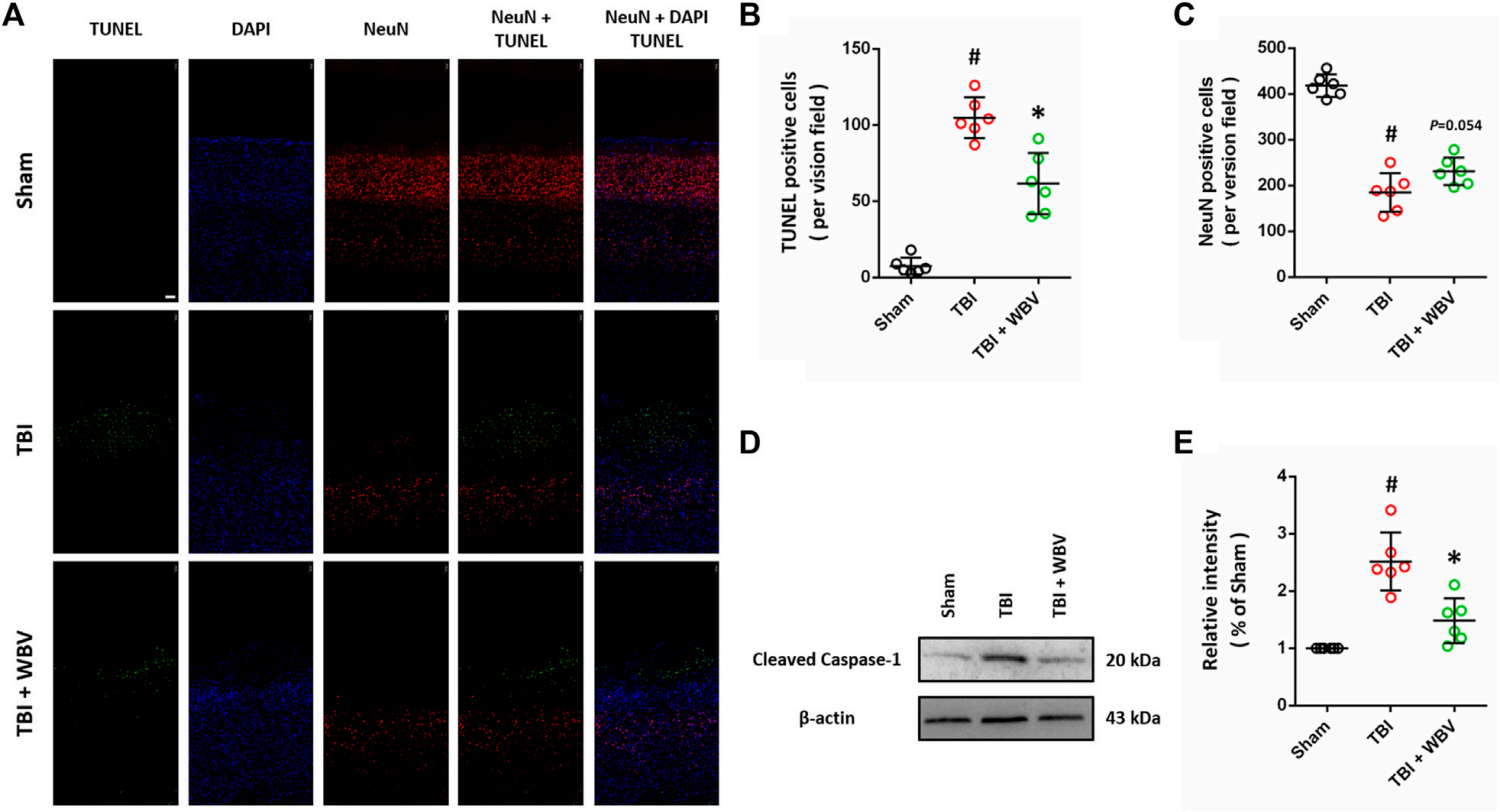
WBV ameliorates neuronal cell death after TBI. **(A–C)** Representative images of TUNEL staining in injured cortex **(A)** and quantitative analysis of TUNEL positive cells **(B)** and NeuN positive cells **(C)**. **(D,E)** Representative images of western blot analysis showing the expression of cleaved caspase-1 (p20) **(D)** and quantitative analysis **(E)**. Scale bar = 100 μm. Data are shown as mean ± SD. ^#^ *p* < 0.05 vs. Sham group. *p < 0.05 vs. TBI group.

TUNEL staining was performed to detect apoptotic cell death in brain sections (Figure 6A), and the results showed that TBI-induced apoptosis was alleviated by WBV (Figure 6B). We also performed immunostaining using NeuN antibody, the molecular marker of healthy neurons, and WBV-treated group have more NeuN-positive cells compared to TBI group (Figure 6C), which is not statistically different (*p* = 0.054). In addition, western blot was performed to detect the cleavage of caspase-1 at 24 h after TBI (Figure 6D), and the results showed that TBI-induced increase in cleaved caspase-1 (p20) was reduced by WBV (Figure 6E).

### WBV Improves Exploratory Behavior and General Activity After TBI

**FIGURE 7:**
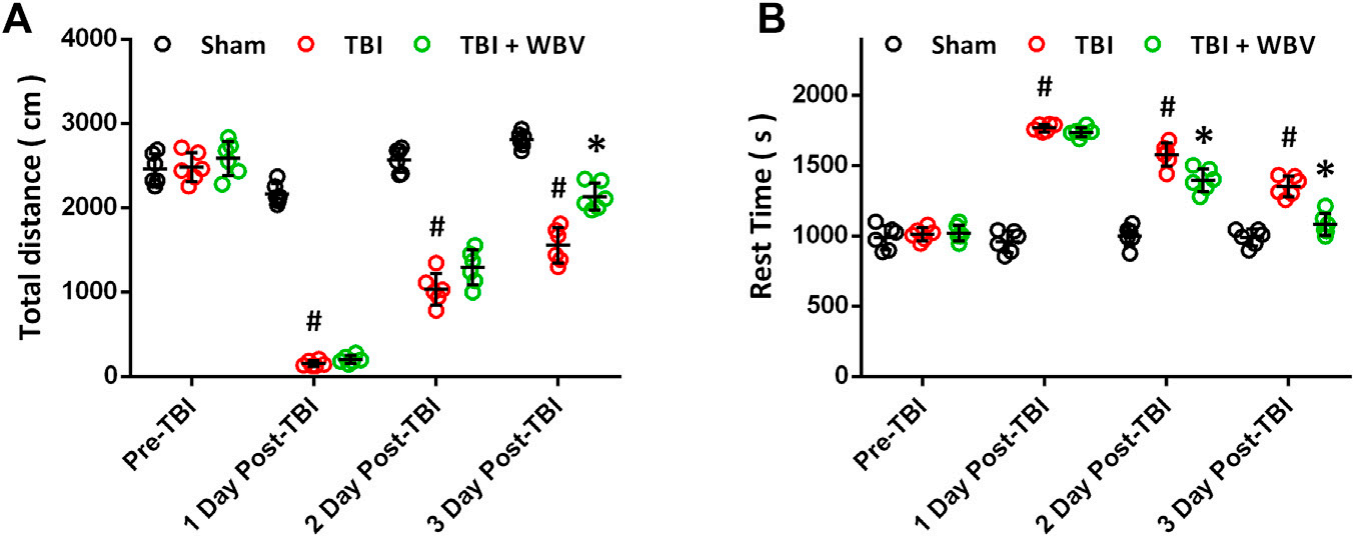
WBV improves exploratory behavior and general activity after TBI. **(A)** Longitudinal assessment of motor activity recovery. **(B)** Side by side longitudinal assessment of rest time. Data are shown as mean ± SD. ^#^ *p* < 0.05 vs. Sham group. *p < 0.05 vs. TBI group.

Then, exploratory behavior (Figure 7A) and general activity (Figure 7B) were regularly recorded to determine the motor activity of the animals. In consistent with previously published data, TBI induced a significant decrease in total distance traveled by the animals and a significant increase in rest time. The mice pretreated with WBV exhibited higher motor activity at 3 days post TBI, as evidenced by the longer total distance traveled by the animals (Figure 7A). Compared to TBI-treated group, the mice pretreated with WBV showed substantially more general activity expressed as less rest time at 2 and 3 days following TBI (Figure 7B).

### WBV Attenuates Learning and Memory Deficits After TBI

**FIGURE 8:**
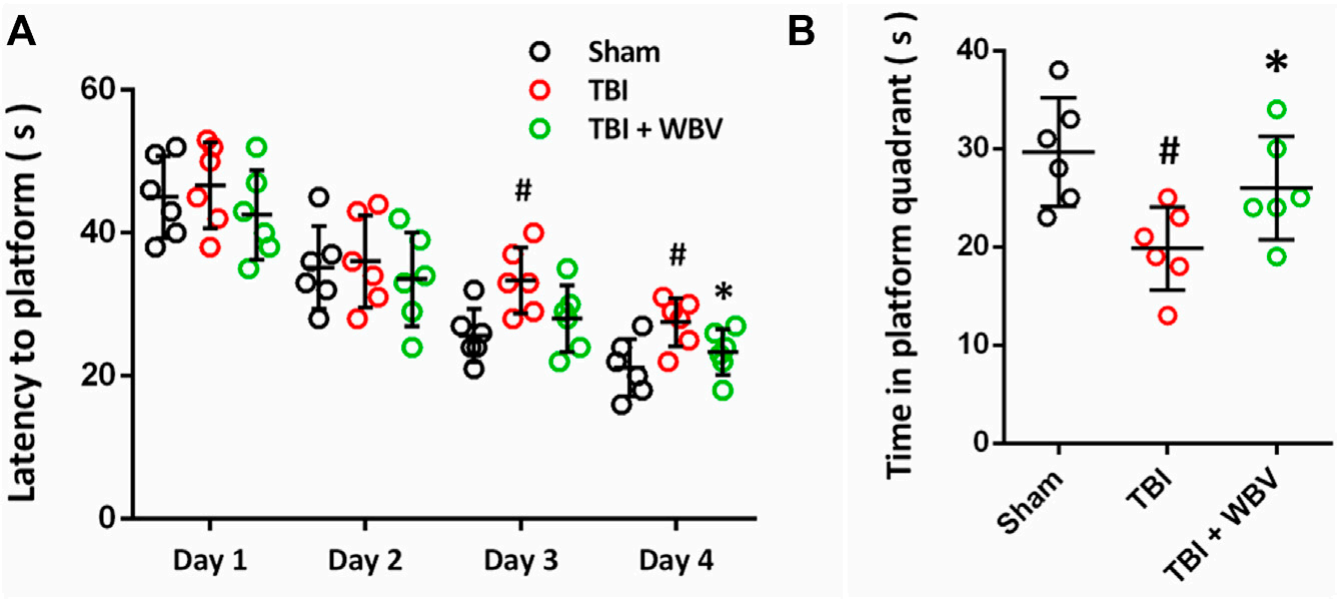
WBV attenuates learning and memory deficits after TBI. **(A)** Learning curves showing the latency to the platform on each day of 4 days of the learning trail. **(B)** Quantitative analysis of time in platform quadrant during the 60 s probe trial. Data are shown as mean ± SD. ^#^ *p* < 0.05 vs. Sham group. *p < 0.05 vs. TBI group.

The effect of WBV on learning and memory deficit caused by TBI was determined by the MWM test (Figures 8A,B). The average latency to platform gradually decreased from day 1 to day 4 (6–9 days after TBI) in all three groups, indicating the learning process. The animals in WBV group showed lower latency to platform compared to TBI group at day 3 and day 4 (Figure 8A), although statistical significance was not reached at day 3 (*p* = 0.08). In the probe test, better performance was recorded in WBV group, indicating the improvement in memory (Figure 8B).

## Discussion

A number of previous studies have been demonstrated that physical exercise is a powerful intervention to improve neurological function in TBI survivors (O'Neil et al., 2019; Vanderbeken and Kerckhofs, 2017). WBV is a novel mimicking exercise that has been used as an alternative to physical therapy in various clinical settings (Park et al., 2018). In this study, we identified WBV treatment, at 30 Hz twice per day for 20 days, as an effective therapeutic approach to protect against experimental TBI in mice. We found that a) WBV alleviates TBI-induced brain edema, without effect on animal body weight; b) WBV suppresses expression of GFAP and Iba-1 after TBI; c) WBV differently regulates inflammatory cytokines; d) WBV ameliorates neuronal cell death after TBI; and e) WBV improves motor and cognitive function following TBI.

WBV can occur in many schedule life scenes, such as riding or driving a vehicle and operating construction machinery. The human body is a dynamic and complex system, and the vibration induced by WBV may induce diverse consequences, depending on the frequency, amplitude and time of exposure (Zhou and Griffin, 2017). It is shown that the mechanical vibration-sensitive receptors, such as Meissner’s corpuscles, are sensitive to the frequencies of vibration between 30 and 40 Hz, and the human body is more sensitive to 1–50 vibration (Firmino et al., 2021). Distinct parts of the body, including chest cavity, abdominal cavity and the brain, resonate under WBV, and 5 Hz vibration could exert more discomfort compared with other frequencies (Liang et al., 2017). In contrast, positive effects of WBV close to 30 Hz were reported by many previous published papers. Regterschot et al. showed that passive WBV (frequency 30 Hz, amplitude 0.5 mm, six sessions) has positive acute effects on cognitive functions in healthy young participants (Regterschot et al., 2014). Fuermaier et al. also found that WBV at 30 Hz significantly improved cognitive performance in both healthy individuals and attention deficit hyperactivity disorder (ADHD) patients (Fuermaier et al., 2014). More importantly, WBV at 30 Hz for 5 weeks was shown to improve cognitive tests performance in both mice and humans (Boerema et al., 2018). In consistent with previous results, our present study found that WBV treatment, at 30 Hz twice per day for 20 days, inhibited brain edema and reduced neuronal cell death after TBI, without effect on animal body weight. In addition, the animals in WBV-treated group have better performance in exploratory behavior, general activity and MWM tasks. These data strongly indicate that WBV with low frequency might be a safe and effective non-pharmacologic intervention for TBI patients.

Previous studies on WBV were mainly focus on movement disorders, as well as its effects on muscle function and balance related to increased muscle strength (Park et al., 2015). Increasing evidence have demonstrated that WBV also has positive effects on other system, such as cardiovascular system, immune system and tissue repair system. Recently, WBV training was demonstrated to be proposed as comprehensive therapy on glycemic control in elderly type 2 diabetes patients (Kitamoto et al., 2021). In addition, the low acceleration WBV at 45 Hz was found to accelerate re-epithelialization, angiogenesis and granulation tissue formation, thereby improving wound healing (Roberts et al., 2021). The authors demonstrated that these effects were associated with the increased levels of insulin-like growth factor (IGF)-1 and vascular endothelial growth factor (VEGF) in the wound. Thus, we speculated that WBV might have positive effects on brain function under neurological diseases *via* direct body-to-brain vibration transmission, or *via* indirect effects on neurotransmitters. A previous study showed that a single 3 min of WBV is sufficient to apparently improve microvascular blood flow (Betik et al., 2021). The increased levels of brain-derived neurotrophic factor (BDNF) and nerve growth factor (NGF) were also observed after WBV in both animals and humans (Kartha et al., 19762014; Simão et al., 2019). In this study, direct positive effects on the brain, as evidenced by reduced brain edema and decreased neuronal cell death, were found after TBI in WBV-treated animals.

TBI is customarily categorized into the primary injury, the external force-induced brain damage itself, and the secondary injury, which is related to various activated detrimental signaling cascades contributing to neurological dysfunction (Krishnamurthy et al., 2016). As one of the most important mechanisms of TBI, neuroinflammation occurs immediately after the primary injury, and participates in many aspects of the secondary injury. TBI-induced neuroinflammation is a sterile inflammatory response that is characterized by the activation of resident glial cells, recruitment of peripheral immune cells, and the release of inflammatory cytokines (Nizamutdinov and Shapiro, 2017; Delage et al., 2021). Following TBI, morphological and functional changes occur within microglia and astrocytes, leading to the disruption of both neuronal-glial and glial-glial interactions (Bal-Price and Brown, 2001). We detected the activation of these glial cells by measuring the expression of their markers, Iba-1 for microglia and GFAP for astrocytes. Our results showed that TBI resulted in increased expression of Iba-1 and GFAP in cortex and hippocampus, and the WBV-pretreated animals showed lower levels of these two proteins, indicating reduced inflammatory response. Intriguingly, the levels of Iba-1 and GFAP in corpus callosum were not altered, which might be explained by that the CCI-induced brain damage is slight in deep structures of the brain, such as corpus callosum. Inflammatory cytokines (both pro-inflammatory and anti-inflammatory) can be secreted by neurons and glial cells, and they exert both detrimental and beneficial effects through regulating the permeability of the blood-brain barrier (BBB) and the recruitment of peripheral immune cells (Kumar and Loane, 2012; Simon et al., 2017). ELISA data from our study showed that the WBV-pretreated animals display lower levels of IL-1β, IL-6 and TNF-α, but higher level of IL-10. These results suggest that regulation of neuroinflammation is involved in WBV-induced protection against TBI.

There are some caveats to this study. First, pretreatment with WBV for 20 days was used before TBI, and this precondition protocol is less relevant to clinical setting, especially for acute TBI. However, it is useful for understanding the effects of WBV on healthy humans, and providing more information for studying post-injury treatment strategy. Some more experiments on the effects of WBV treatment after TBI awaits further study. In addition, the frequency and amplitude were controlled by measuring at the surface of the vibrating plate, but the actual vibration parameters experienced by the brain cannot obtained here. More advanced testing equipment are needed, and a vibrator directly attached to the animals’ head might provide us more information of the changes of the brain under WBV.

In summary, this study demonstrated that WBV pretreatment at 30 Hz for 20 days markedly reduced brain damage and improved functional and cognitive outcomes after TBI. These positive effects were related to its effects on neuroinflammation and apoptosis of neuronal cells. In view of these results, the low frequency WBV might be an ideal non-pharmacologic intervention for TBI patients.

## Data Availability

The original contributions presented in the study are included in the article/Supplementary Material, further inquiries can be directed to the corresponding authors.
