# Supplementary figures and images for: Whole Body Vibration Attenuates Brain Damage and Neuroinflammation Following Experimental Traumatic Brain Injury
Source: Front Cell Dev Biol. 2022 Apr 7;10:847859. doi: 10.3389/fcell.2022.847859 (PMC9022659; doi:10.3389/fcell.2022.847859)

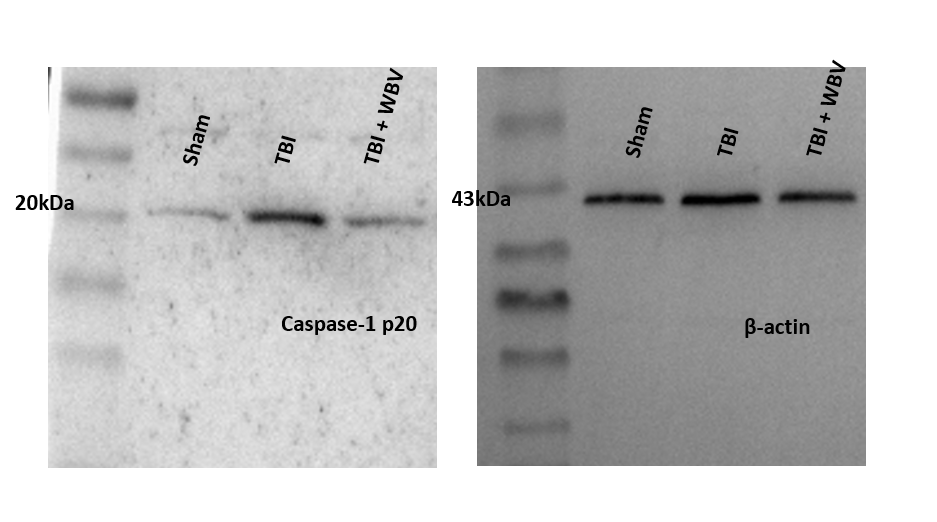

Supplement: Supplementary file 1 [file Image1.TIF]
